# Between shame, control, and survival: a grounded theory study of eating disorders among young Chinese women

**DOI:** 10.1186/s40337-025-01510-9

**Published:** 2026-03-31

**Authors:** Qianhui Tang, Jingying Jiang, Shuhui Fan

**Affiliations:** Faculty of Psychology, Beijing Normal University, Beijing, China

**Keywords:** Eating disorders, Chinese women, Grounded theory, Emotional regulation, Sociocultural factors, Qualitative research

## Abstract

**Background:**

Eating disorders (EDs) among young Chinese women are shaped by sociocultural norms and moralized expectations around thinness and emotional restraint, yet theory grounded in local experiences remains limited.

**Method:**

Using constructivist grounded theory, we conducted semi-structured interviews with 19 women with current or past ED experiences. Data were analyzed iteratively (open–focused–theoretical coding) with constant comparison, sending analytic memos, and team debriefing to build an explanatory model.

**Results:**

Four interrelated themes were identified: (1) risk background, including personality traits, adverse developmental experiences, and sociocultural pressures; (2) onset pathways—appearance-driven and emotion-driven routes; (3) maintenance and change processes, shaped by shame, functional reinforcements, and social feedback; and (4) coping strategies, including professional help, social support, and self-directed adjustment. Thinness was moralized as a symbol of discipline and social worth, while shame reinforced secrecy and chronicity. Turning points often arose through autonomy-supportive relationships and growing awareness of harm.

**Discussion:**

This study expands existing models by theorizing how EDs are embedded in the Chinese sociocultural context. It highlights the moralized nature of body control and emotional suppression in shaping illness experiences. Clinical implications include the need for shame-sensitive, culturally attuned interventions.

**Supplementary Information:**

The online version contains supplementary material available at 10.1186/s40337-025-01510-9.

## Abstract (plain language summary)

This study explored how eating disorders develop and persist among young women in China. We interviewed 19 women who had experienced eating problems to understand what these struggles meant in their everyday lives. The findings show that eating disorders often begin along two intertwined routes: one appearance-driven, the other emotion-driven. Many participants described how eating behaviors—restricting, bingeing, or purging—helped them cope with pain, regain control, or seek approval from others. In Chinese families and social circles, being slim was often seen as a sign of discipline and moral worth, while weight gain was viewed as personal failure. Shame was a powerful emotion that kept many women silent and isolated. Recovery became possible when they encountered empathy, autonomy, and genuine understanding. This study highlights the need for culturally sensitive, shame-aware approaches that help individuals reconnect with self-worth beyond body control.

**Supplementary Information:**

The online version contains supplementary material available at 10.1186/s40337-025-01510-9.

## Introduction

Eating disorders (EDs) are a group of severe psychiatric conditions characterized by persistent disturbances in eating or related behaviors. They carry the highest mortality rate among psychiatric illnesses and contribute substantially to the global burden of disease, particularly among young women [6, 40]. Global prevalence has nearly doubled in recent decades, now affecting around 7–9% of the population [27, 59]. In China, national data show that more than 20% of adolescents screen positive for probable EDs [7], underscoring the urgency of culturally informed understanding. Physiologically, EDs lead to malnutrition, electrolyte imbalance, and multisystem complications [66]. Psychologically, EDs co-occur with depression, anxiety, and suicidality [60, 68], while socially, affected individuals often experience stigma, isolation, and strained relationships [31, 42]. Long-term studies reveal slow, relapse-prone recovery—only one-third of anorexia nervosa patients recover within nine years, while over 60% do so after two decades; nearly 40% remain symptomatic and one-quarter relapse in early adulthood [19, 23]. Collectively, these findings underscore the chronic, systemic nature of EDs and the need for integrative theoretical and clinical approaches.

Decades of research have yielded influential frameworks to explain ED onset and maintenance. Sociocultural models emphasize the internalization of unrealistic body ideals and appearance-based social comparison [61, 62], transdiagnostic models identify shared cognitive-behavioral mechanisms across various ED subtypes [22]; and emotion regulation theories conceptualized disordered eating as a maladaptive strategy for managing affect [30, 37]. However, these paradigms were developed primarily within white, Western, middle-class populations [64], raising concerns about cross-cultural applicability. While EDs occur globally, their sociocultural expressions and etiological pathways differ substantially, with women disproportionately affected [8, 51].

In contemporary China, EDs arise within a unique sociocultural landscape. The rising prevalence among young women [43] coincides with collectivist values, gendered expectations of restraint, and hierarchical family relationships that intersect with modern appearance ideals [31, 42]. Within this context, thinness is closely tied to social success, moral discipline, and familial approval, intensifying pressure on women to conform to restrictive beauty norms [41]. Qualitative studies reveal pervasive body surveillance and evaluative commentary from family, peers, and media, reflecting how appearance becomes a moral and relational concern [31]. These dynamics not only precipitate EDs but also foster emotional suppression and secrecy, reinforcing illness chronicity.

While sociocultural pressures lay the groundwork for disordered eating, the psychological mechanisms through which they operate reveal deeper cultural dynamics. Beyond appearance concerns, EDs in China often function as coping strategies for emotional distress, family expectations, and unmet psychological needs [28, 31]. These pressures are internalized through moral emotions such as shame and self-restraint, reflecting the Confucian emphasis on modesty, filial piety, and self-discipline [67, 69]. Rather than being driven primarily by the pursuit of an individualistic thin ideal—central to Western frameworks such as objectification and sociocultural internalization models [26, 62]—Chinese experiences of disordered eating may be infused with relational and moral meanings. Shame, directed toward both the body and perceived behavioral failure, operates as a powerful emotional driver of secrecy and avoidance [42]. In a broader social context, stigma surrounding mental illness and cultural norms discouraging emotional disclosure further reinforce the somatization of distress [1]. Taken together, these dynamics highlight the limitations of applying Western-derived models to Chinese populations and underscore the need for culturally attuned frameworks that incorporate moral emotions, relational interdependence, and embodied expressions of suffering.

Although a growing body of work has begun to illuminate the cultural dynamics of EDs in Asia, the literature remains fragmented. Existing studies have examined cultural tensions around food and body [48], media influences on body dissatisfaction [32], and perfectionism as a maintenance factor in Chinese ED patients [71]. Yet few have offered an integrative theoretical account of how sociocultural norms and psychological processes interact to shape illness trajectories. Grounded theory offers a powerful approach to address this gap, generating data-driven models that capture lived complexity. Prior grounded theory studies have illuminated relational and systemic processes such as partner adaptation [49] and family reorganization [34], but none have systematically theorized how young Chinese women experience, sustain, and manage EDs.

Accordingly, this study adopts a constructivist grounded theory approach to explore how EDs are experienced, maintained, and managed by young Chinese women. Specifically, it seeks to address three guiding questions: (1) How are EDs generated in the lived experiences of young Chinese women? (2) Why and how do EDs persist, becoming entrenched psychological structures despite their harmful consequences? (3) What coping strategies are adopted by young Chinese women with EDs, and which of these are perceived as effective or ineffective in managing the ED?

By situating women’s narratives within their sociocultural context, this study aims to construct a theoretical model that links cultural, emotional, and relational mechanisms in ED development. The findings seek to enrich both localized understanding and global ED theories by introducing culturally situated explanatory pathways.

## Methodology

### Design and rationale

This study employed a constructivist grounded theory (CGT) approach [15] to explore how EDs are experienced, maintained, and managed among young Chinese women. CGT emphasizes the co-construction of meaning between researchers and participants, allowing theory to emerge inductively from participants’ lived experiences. Given the culturally embedded nature of EDs and the limited body of localized research in China, this approach was well-suited to uncover the nuanced sociocultural and emotional processes underlying EDs.

Semi-structured interviews were used to enable in-depth exploration while maintaining flexibility for participants to share personally meaningful narratives. This format ensured both thematic coherence and openness to emergent issues, consistent with CGT’s iterative and discovery-oriented design.

### Participants and sampling

We recruited nineteen self-identified Chinese women aged 18–31 who had received a clinical diagnosis of an ED from qualified mental health professionals. Recruitment was conducted through university-based mental health platforms, social media posts, and snowball sampling. In the initial phase, purposive sampling prioritized information-rich cases—such as early onset, longstanding illness duration, or co-occurring psychological conditions—to explore diverse illness trajectories. As the analysis progressed, theoretical sampling guided the inclusion of participants with varying diagnoses, symptom severity, and recovery status to enrich conceptual categories [15]. No prior relationship existed between the researchers and any of the participants.

Five participants took part in pilot interviews to refine the semi-structured interview guide and ensure the cultural and linguistic appropriateness of key questions. These women were aged between 21 and 28, all had been formally diagnosed with anorexia nervosa, bulimia nervosa, or binge-eating disorder, and two reported co-occurring conditions such as depression or obsessive–compulsive disorder. Most were single, and their highest educational attainment ranged from undergraduate to master’s level. The pilot interviews provided preliminary insights into experiences of shame, control, and emotional distress, which informed the design of the main study but were not included in the final analysis.

The remaining fourteen participants, aged 19–31, were included in the main analysis. Their clinically confirmed diagnoses included anorexia nervosa, bulimia nervosa, and binge-eating disorder, with several participants experiencing overlapping symptoms or co-occurring mood and anxiety disorders. Most were unmarried, and their highest educational attainment ranged from undergraduate to master’s level. Participants came from a range of provinces across China, reflecting diverse sociocultural and family backgrounds. The duration and onset of illness varied from early adolescence to adulthood, and their recovery statuses ranged from ongoing treatment to sustained remission. This heterogeneity enriched the theoretical sampling and deepened the understanding of the sociocultural and emotional dynamics surrounding eating disorders among young Chinese women.

### Data collection

We conducted one-on-one semi-structured interviews via secure video conferencing to ensure privacy and accessibility across regions. Each session lasted 60–90 min, and no non-participants were present during the interviews, ensuring a private and safe environment for participants to share their experiences. Field notes were taken immediately after each interview to capture the interviewer’s observations and reflections, which later informed analytic memo-writing. Data collection continued until theoretical saturation was reached. After twelve formal interviews, no new conceptual categories emerged; two additional interviews were conducted to confirm saturation [29].

Guided by the research aims and grounded theory methodology, the interview guide was developed by the research team based on existing literature and clinical insights into EDs, and iteratively refined through five pilot interviews to ensure clarity, emotional safety, and thematic depth (see Additional file 2). The guide covered four major domains—illness onset, maintenance mechanisms, sociocultural and relational influences, and coping or recovery strategies. All interviews were audio-recorded with consent, transcribed verbatim in Chinese, and anonymized for analysis.

Given the emotional sensitivity of the topic, a crisis response protocol was established in advance. The interviewer was a graduate student in clinical and counseling psychology with prior experience in counseling and crisis intervention. She was prepared to pause or terminate interviews if participants showed signs of distress and to provide supportive responses when needed. Participants were also provided with a list of local mental health resources at the end of each interview. No acute incidents occurred during data collection.

### Data analysis

Data were analyzed using Charmaz’s [15] constructivist grounded theory framework, emphasizing iterative engagement and the co-construction of meaning. Analysis proceeded through four stages: initial, focused, axial, and theoretical coding, supported by NVivo 12 software for data management.

During initial coding, transcripts were examined line by line using short, action-oriented codes in gerund form (e.g., “restricting eating,” “seeking approval”) to stay close to participants’ voices. Focused coding grouped recurring patterns into broader conceptual categories. Axial coding explored relationships among these categories—conditions, strategies, and consequences—through constant comparison across cases. Finally, theoretical coding integrated the main categories into a coherent model explaining the emergence, maintenance, and coping processes of EDs. 

*Coding team and consensus* The first author conducted the primary coding of all interview transcripts and engaged in extensive memo writing throughout the process. The second author independently coded the majority of transcripts to enhance interpretive depth and consistency. Coding discrepancies were thoroughly discussed and resolved through consensus meetings under the supervision of the senior author (SF), who provided ongoing guidance and critical feedback to ensure theoretical coherence and reflexive rigor. This collaborative analytic process strengthened the credibility of the findings and supported the development of a contextually grounded theoretical model.

Throughout the analysis, memo writing documented reflections and analytic decisions, and regular team debriefings were held to refine interpretations and ensure that findings remained grounded in participants’ narratives.

### Trustworthiness and rigor

Reporting adhered to the COREQ (Consolidated Criteria for Reporting Qualitative Research) checklist to ensure transparency and completeness of qualitative reporting (see Additional file 1). To ensure the trustworthiness of this study, multiple strategies were employed in accordance with established qualitative research standards [39, 58]. These strategies addressed credibility, dependability, confirmability, and transferability.

*Triangulation* Investigator triangulation was achieved through collaborative review of coding decisions and continuous analytic discussions within the research team [14]. Theoretical triangulation was further applied by comparing emerging findings with relevant literature to refine conceptual understanding while avoiding the premature imposition of external frameworks.

*Peer debriefing* Regular debriefing sessions were held within the research team and with external qualitative scholars to promote reflexivity, challenge assumptions, and enhance analytic rigor [16]. These dialogues provided an external perspective and strengthened the credibility of the interpretations.

*Member checking* Three participants representing different diagnoses and illness stages were invited to review thematic summaries and comment on their accuracy and resonance. All confirmed that the findings reflected their lived experiences, reinforcing the credibility and authenticity of the results [10].

*Audit trail and reflexivity* An audit trail was maintained throughout the study, including documentation of recruitment procedures, coding decisions, memo-writing, and theory-building. This trail allows for transparency and external verification of the analytical pathway [70]. The inclusion of reflexive journals helped monitor potential researcher bias, contributing to the confirmability of the study [24].

*Dependability and transferability* To enhance dependability, all analytical decisions were reviewed by the supervisory researcher for consistency. In-depth description of participants’ contexts and experiences was provided to facilitate transferability and allow readers to evaluate the applicability of findings to other settings.

### Reflexivity statement

As female researchers trained in clinical and counseling psychology, we approached this study with both professional insight and cultural attunement. The first author had prior counseling experience with individuals facing body image and eating-related concerns and had also personally experienced periods of disordered eating in the past. These experiences deepened her empathy and understanding of participants’ struggles but also required heightened reflexivity to prevent overidentification. Regular supervision and peer debriefing were incorporated throughout the research process to sustain reflexive awareness and maintain balance between emotional resonance and analytical distance. We acknowledge that our interpretations were inevitably shaped by our sociocultural positions and lived experiences within Chinese academic and therapeutic contexts, and we embraced this subjectivity as an integral part of the co-constructive process of grounded theory research.

### Ethical considerations

This study was approved by the Ethics Committee of the Faculty of Psychology, Beijing Normal University. All participants provided informed consent, were assured of confidentiality and voluntary participation, and were informed of their right to withdraw at any time. Pseudonyms were used in transcripts and reports to protect participants’ identities.

## Results

Through constructivist grounded theory analysis of interviews with 19 participants, this study developed a theoretical framework detailing the emergence, development, and coping processes of eating disorders (EDs) among young Chinese women. Four core themes were identified: (1) Risk Factors, (2) Pathways of Onset, (3) Maintenance and Change, and (4) Coping Strategies.

### Risk factors

Specific risk factors were identified that contribute to the likelihood of developing EDs among young Chinese women: (1) individual personality traits, (2) adverse developmental experiences, and (3) socio-cultural pressures (see Table 1). These factors represent an interplay between internal vulnerabilities and external environments, laying the foundation for disordered eating patterns.

**Table 1:** Risk factors of EDs

Subcategory	Focused code	Number of participants	Frequency
Personality Traits	Perfectionism	6	10
	Low Self-Esteem	7	13
	Sensitivity to Others’ Evaluations	5	12
	Interpersonal Distrust	10	20
Adverse Developmental Experiences	School Bullying and Exclusion	5	8
	Body Shaming and Humiliation	6	20
	Traumatic Events	3	3
	Family Relationship Difficulties	10	30
Socio-cultural Environment	Societal Beauty Standards	9	13
	Gender-Specific Pressure	6	9

This table presents the main risk factors contributing to the onset of eating disorders, categorized into personal traits, adverse experiences, and sociocultural influences. The number of participants referencing each focused code and the frequency of its occurrence in interviews are listed

#### Personality traits

Participants frequently linked their EDs to enduring personality characteristics that heightened vulnerability. Four traits were especially prominent: perfectionism, low self-esteem, sensitivity to external evaluation, and interpersonal distrust.

*Perfectionism* Perfectionism was cited as a driving force behind rigid self-demands, particularly around appearance and weight control. Several participants framed disordered eating as an extension of their pursuit of flawlessness. As one put it,

*“It’s because you have this pursuit of perfection that you choose to engage in such behaviors. Without it*,* you wouldn’t feel the need to be thin or particularly good-looking*,* so you wouldn’t fall into the cycle of eating disorders so easily” (P1).*

*Low self-esteem* Many interviewees noted that their low self-esteem fueled their compulsive need to adhere to strict standards, believing that meeting these self-imposed rules would help boost their self-worth. One participant reflected:

*“I felt inferior to others*,* and that led me to establish compulsive rules for myself. It wasn’t recognized or addressed*,* and it came back as an eating disorder in college” (P8).*

*Sensitivity to others’ evaluations* Participants described being highly sensitive to the opinions of others, especially regarding their appearance. This sensitivity frequently led them to engage in weight control behaviors to gain positive social feedback.

*Interpersonal distrust* Distrust of others, particularly in terms of emotional support, was also a common theme. Many participants described handling their eating issues privately, without seeking help, due to their belief that others wouldn’t understand or offer meaningful assistance.

Together, these personality-related risk factors reveal how enduring internal vulnerabilities—such as perfectionism and shame-prone self-concept—can interact with social feedback to create fertile ground for disordered eating to emerge and persist.

#### Adverse developmental experiences

A range of distressing experiences during childhood and adolescence contributed to participants’ emotional vulnerability and body dissatisfaction. These included peer bullying, body-related humiliation, trauma, and difficult family dynamics.

*School bullying and exclusion* Many participants described being bullied or excluded by peers during their school years. These experiences of rejection and marginalization undermined their self-worth and increased their emotional vulnerability. Lacking supportive figures, participants reported turning to food—either as a source of comfort or as an area where control could be exerted amid social chaos.

*Body shaming and humiliation* Critical or mocking comments about participants’ bodies—often from family members, peers, or romantic partners—were frequently cited as formative experiences. These moments of humiliation appeared to embed body-related anxiety and drive a persistent dissatisfaction with one’s physical self. For some, these comments catalyzed the onset of disordered eating behaviors; for others, they exacerbated pre-existing insecurities. As one participant shared:

*“I gained weight around the age of 15*,* and my father had a big influence on me. I was eating*,* and he just said*,* ‘Don’t eat anymore…’ He didn’t want me to get fat*,* and his words left a deep mark on me. I still remember what he said—telling me not to eat” (P14).*

*Traumatic events* A subset of participants experienced major trauma, including sexual assault and bereavement. One woman, whose disclosure of abuse was met with disbelief from her mother, described a persistent sense of emotional unsafety that shaped her eating patterns. She noted that this distress lasted for a long time—“*I was in this state for a long period where if my stomach wasn’t full*,* I would feel very anxious and felt I had to keep eating*”*(P4)*. For her, hunger sensations quickly triggered emotional collapse, and eating offered rapid bodily relief rather than pleasure. The lack of validation after trauma intensified feelings of betrayal, loneliness, and internal tension, leaving her with few relational avenues for support. In the absence of emotional safety, disordered eating became one of the only predictable ways she could soothe distress and re-establish a fragile sense of control. These accounts underscore how trauma can impair both emotional regulation and help-seeking, reinforcing eating behaviors as a self-contained form of control and relief.

*Family relationship difficulties* Several participants reported significant negative experiences within their families, including chronic conflict, emotional neglect, and critical or controlling parenting. These stressful dynamics often undermined emotional safety and hindered the development of self-worth and regulation skills. In such environments, disordered eating became a way to manage distress and assert autonomy, structure, and control amid relational chaos.

Across participants’ narratives, adverse developmental experiences undermined emotional safety and self-worth, laying the groundwork for disordered eating to emerge as a coping strategy in the absence of supportive relationships.

#### Socio-cultural environment

The broader socio-cultural environment—particularly prevailing aesthetic norms and gendered expectations—was identified as a major contextual factor in the emergence of EDs. Participants described a social climate saturated with thinness ideals, where beauty is narrowly defined and relentlessly promoted through media, peer influence, and familial messaging. These external pressures were deeply internalized and frequently shaped participants’ sense of self-worth and behavioral choices.

*Societal beauty standards* Across narratives, participants expressed that thinness is not merely preferred but socially mandated, especially for young women in contemporary Chinese society. This cultural valorization of slenderness, often intertwined with moral judgments of discipline, self-control, and success, created intense pressure to conform.

*“No one ever taught me a body-positive way of seeing myself. Most people only accept you when you’re slim and pretty—and they want you to stay that way. If I gained weight*,* I didn’t even dare go outside”* (P14).

*Gender-specific pressures* Participants noted that aesthetic standards were far more stringently applied to women. Their bodies were constantly scrutinized, not only in public but also in intimate and familial settings. Appearance became a key domain of feminine social performance, and deviation from thinness often invited criticism, dehumanization, ridicule, or pity.

One participant reflected on the influence of online culture during her adolescence:

*“When we were younger*,* there was this online forum called ‘Good Girls Don’t Weigh Over 100.’ It was all like*,* ‘How could you weigh over 100 pounds? You’re basically a pig!’ Posts like that were everywhere. I was over 100 pounds at the time and thought*,* ‘What now? Should I just disappear or kill myself?’ I was joking*,* but I was so anxious. How could people say that being over 100 pounds means you’re not even human?”* (P5).

Such messages, especially during adolescence, provoked lasting shame and anxiety.

Together, participants’ narratives reveal how socio-cultural ideals—amplified by media, peers, and family—impose narrow standards of thinness and femininity, reinforcing body surveillance and psychological distress that lay the groundwork for EDs.

### Onset pathways

Participants described two primary trajectories through which their EDs developed: one centered on weight-related concerns and the other rooted in emotional distress and stress-related coping (see Table 2). These pathways were not mutually exclusive and often overlapped over time.

**Table 2:** Onset pathways

Subcategory	Focused code	Number of participants	Frequency
Weight Concern and ED	Weight-Control Attempts	11	24
	Intense Fear of Weight Gain	6	34
	Rigid Diet Control	9	27
	Binge Eating following Food Cravings	8	21
	Feelings of Guilt and Compensatory Behaviors	7	13
Stress, Emotions, and ED	Stressful Life Events	8	23
	Emotional Distress	9	19

This table summarizes the two primary onset pathways identified in participant narratives: one centered on weight-related concerns and the other on emotional distress. The number of participants referencing each focused code and the frequency of its occurrence in interviews are listed

#### Weight concern and ED

The first pathway highlights a progression from body image dissatisfaction to disordered eating behaviors. This trajectory commonly unfolded in the following sequence: (1) weight-control attempts, (2) fear of weight gain, (3) rigid dietary control, (4) binge eating following food cravings, and (5) guilt and compensatory behaviors.

*Weight-control attempts* Many participants traced the onset of their EDs to early efforts to change their body shape. These motivations varied—some were influenced by media or beauty norms, others attributed relationship problems (e.g., poor treatment by others) and difficulties with self-esteem to their appearance.

*“I started dieting because I thought being thinner might fix how people treated me. It wasn’t just about looks—it felt like the problem was my body”* (P11).

*Intense fear of weight gain* Alongside these efforts emerged a pervasive fear of gaining weight, which fueled restrictive eating and purging behaviors. Participants described obsessive thoughts and extreme anxiety about any perceived weight fluctuation.

*“If it came down to choosing between being fat and starving myself to death*,* I might really choose to starve myse*lf” (P5).

*Rigid diet control* Driven by these fears, interviewees began strict dietary control, including strict calorie limits and food classification systems.

*“I had a blacklist and a whitelist for food—these I can eat*,* those I can’t. I wouldn’t allow myself”* (P8).

*Binge eating following food cravings* Several interviewees described an intense craving for food after strict dietary control. This craving could become uncontrollable, consuming their attention and energy and even affecting daily life. When the craving for food cannot be satisfied or controlled, individuals might engage in binge eating.

*“At one point I couldn’t study*,* couldn’t do anything. I was just thinking about food all day*,* scrolling through pictures of food online. I really wanted to eat”* (P13).

*Feelings of guilt and compensatory behaviors* Binge episodes were typically followed by guilt and compensatory behaviors, such as purging or over-exercising, to ‘undo’ perceived damage and relieve distress.

*“The first time I binged*,* I didn’t eat at all the next day—just drank water. But soon*,* I couldn’t control my appetite anymore. After binging*,* I’d make myself throw up*,* then go run 10 kilometers and work out for three more hours*,* trying to undo the damage”* (P13).

#### Stress, emotions, and ED

The second onset pathway highlights the role of external stressors and emotional dysregulation. Participants described developing disordered eating in response to overwhelming life stress and unmanageable emotions, often using food as a coping mechanism.

*Stressful life events* Many participants reported that academic pressure, interpersonal conflict, or major life transitions preceded the onset of their symptoms. Some turned to food for comfort, while others responded with rigid control as a way to regain a sense of agency.

*Emotional distress* Difficult emotions—particularly loneliness, boredom, or sadness—were common triggers for binge eating. Lacking effective emotional regulation strategies, participants relied on food to self-soothe.

*“I tend to binge when I’m feeling lonely or bored—when I don’t want to work or study*,* and I don’t have a good way to entertain myself. Eating makes me feel better*,* at least for a little while”* (P8).

These accounts demonstrate that, beyond concerns about appearance, eating disorders can serve as maladaptive strategies for managing psychological distress and unmet emotional needs.

### Development: maintenance, deterioration, and change

This theme explores how EDs are sustained, how they intensify, and what eventually prompts change. Subthemes include the functions and benefits of EDs, stigma, negative impacts, and factors contributing to deterioration, detailed in Table 3.

**Table 3:** Development: maintenance, deterioration, and change

Subcategory	Focused code	Number of participants	Frequency
Perceived Functions and Benefits of ED	Positive Social Feedback	8	30
	Emotional Regulation and Stress Management	10	31
	Sense of Control, Achievement, and Confidence	7	12
	Satisfaction of Eating Desires Without Concerns About Weight Gain	6	7
Stigma and Shame	Internalized Shame and Global Self-Loathing	9	16
	Dual Identity as Psychological Conflict	3	9
	Behavioral Concealment Driven by Shame	9	27
	Anticipated Rejection and Social Withdrawal	7	20
Negative Impacts of EDs	Physical Health Deterioration	13	39
	Psychological Distress	9	33
	Social Withdrawal and Isolation	9	35
	Academic and Occupational Impairment	10	21
Factors Contributing to Deterioration	Interpersonal Conflicts	8	17
	External Stressful Events.	6	16
	Social Misunderstanding and Denial.	8	19
	Limited Mental Health Literacy	8	13
Turning Point: Emergence of Motivation for Change	Recognition of Harm and Loss	7	18
	“I Don’t Want to Live Like This Anymore”	5	9
	Receiving Acceptance and Support from Others.	6	8

This table presents key themes related to the development of eating disorders, including factors that maintain or worsen the condition and experiences that motivated participants to seek change. The number of participants referencing each focused code and the frequency of its occurrence in interviews are listed

#### Perceived functions and benefits of ED

Based on the interviewees’ accounts, EDs serve specific functions and provide certain benefits in individuals’ lives, which complicates their ability to abandon or modify these behavior patterns, thereby perpetuating the disorder. As one participant noted,

*“People are driven to seek benefits and avoid harm. If something brought only pain*,* I wouldn’t keep doing it. Therefore*,* EDs offer unique advantages that I cannot relinquish*,* which is why I keep doing it”* (P2).

Analysis of the interview data reveals that the functions and benefits of EDs can be categorized into four main areas.

*Positive social feedback* Many participants reported that initial weight loss led to noticeable changes in how others treated them—receiving compliments, admiration, and even romantic attention. These affirming responses made them feel valued and validated, reinforcing their focus on appearance and strengthening the motivation to maintain or intensify restrictive behaviors.

*“When I lost weight*,* people started treating me differently. Even those who used to tease me stopped. They would say things like*,* ‘Wow*,* you’re so skinny now!’… It felt absurd but good”* (P5).

*Emotional regulation and stress management* Several participants viewed binge eating or purging as coping tools that helped manage distressing emotions. In some cases, these behaviors were even described as protective—substituting for other destructive actions such as self-harm.

*“Maybe the only good thing about it was that I hurt myself less. I binged and purged instead of doing worse things to myself when I was falling apart”* (P4).

*Sense of control, achievement, and confidence* Controlling diet and weight can provide individuals with a newfound sense of control, achievement, and confidence, making it challenging to relinquish their control over eating and weight.

*“When I was dieting*,* I thought I was amazing. It was the only thing I could feel proud of—something I could beat others at”* (P8).

*Satisfaction of eating desires without concerns about weight gain* Participants perceive the functional aspect of ED behaviors as a way to satisfy their eating desires through purging or restrictive practices without the concern of weight gain.

These functional benefits played a key role in the persistence of ED behaviors.

#### Stigma and shame

Shame emerged as a deeply pervasive theme throughout participants’ narratives, reinforcing the secrecy and persistence of disordered eating. The following four categories capture how shame shaped participants’ experiences and contributed to the maintenance of eating disorders.

*Internalized shame and global self-loathing* Participants often described their disordered behaviors not simply as unhealthy, but as morally reprehensible. They interpreted bingeing, purging, or loss of control as personal failures or character flaws, contributing to persistent self-condemnation.

*“To my parents*,* I’m just punishment—payback. I’m a burden… If everyone had keywords to describe their life*,* mine would be ‘greasy*,*’ ‘sewer.’ That’s who I am”* (P2).

This sub-theme highlights how moralized self-perceptions became tightly intertwined with ED behaviors. Several participants described a cycle in which disordered eating was experienced as evidence of their ‘moral failure,’ reinforcing the very identities they struggled against. To reduce the cognitive dissonance between their negative self-beliefs and their actions, they enacted behaviors that further consolidated these self-defeating identities. Addressing these implicit moral judgments may therefore be a key target for culturally sensitive ED interventions for Chinese women.

*Dual identity as psychological conflict* A recurring motif was the experience of split selfhood—a socially acceptable facade and a hidden, shame-ridden self engaged in secret eating rituals. The tension between these identities created confusion, disgust, and deep emotional pain.

*“I looked polished and perfect outside. But when I binged*,* I felt so filthy*,* so disgusting—like a rat sneaking food in the dark. I couldn’t let anyone see that part of me”* (P13).

This sub-theme illustrates the internal pressure and psychological conflict produced by holding a shame-ridden self alongside a socially acceptable façade. Several participants described feeling caught between sociocultural expectations of perfection and collectivist ideals of maintaining harmony, and their own authentic needs and sense of selfhood. These dualities intensified their internal struggle and contributed to reluctance in seeking support. Recognition of these tensions—and helping women develop strategies to navigate them—may be important for culturally responsive ED interventions for Chinese women.

*Behavioral concealment driven by shame* Many participants reported engaging in elaborate efforts to hide their disordered eating behaviors, including storing food secretly, avoiding shared meals, and purging in private. Such behaviors were shame-driven and rooted in a deep fear of judgment, further isolated individuals.

*“Everything had to be done in secret—eating*,* vomiting*,* hiding food. I can’t explain what it felt like*,* always having to avoid people’s eyes”* (P12).

*Anticipated rejection and social withdrawal* Participants often assumed that disclosing their illness would result in rejection, ridicule, or abandonment. This anticipation of negative responses inhibited help-seeking and led to progressive social withdrawal, particularly in food-related settings.

These narratives show that shame is not just emotional discomfort but a profound identity threat. It discourages disclosure, sustains secrecy, and intensifies psychological distress—functioning as a core maintaining force in the disorder rather than a mere consequence.

#### Negative impacts of EDs

Participants described the extensive and long-lasting toll of EDs on their physical health, psychological well-being, social functioning, and academic or occupational performance.

*Physical health deterioration* Common symptoms included fatigue, muscle weakness, digestive issues, menstrual irregularities, and dental problems—often linked to malnutrition and purging. Several participants noted a marked loss of physical capacity in daily life:

*“In middle school*,* I could jump over two meters. Now in college*,* I barely reach 1.4. My legs feel like dried branches—no strength at all. I get out of breath just climbing stairs”* (P2).

*Psychological distress* Participants reported emotional instability, anhedonia, and obsessive focus on food and body image. Many described losing interest in daily life and experiencing emotional numbness, irritability, or despair.

Intrusive thoughts about weight and eating often overwhelmed their mental space, fueling anxiety and self-loathing:

*“I would feel so anxious and panicked every time I lost control and ate too much. The fear of getting fat would just overwhelm everything else”* (P13).

*Social withdrawal and isolation* EDs disrupted participants’ social lives, particularly around food-related settings. Many avoided eating in public or declined social invitations due to fear of judgment, the need to maintain control, or the inability to engage in compensatory behaviors afterward. As one noted,

*“I didn’t want to eat with anyone. I didn’t want them to see how I ate or try to stop me from losing weight. I just wanted to be left alone”* (P5).

*Academic and occupational impairment* The disorder consumed substantial cognitive and emotional resources, impairing participants’ focus and task performance. Many reported declines in academic or work functioning due to the time spent managing food, weight, and compensatory behaviors.

*“I spent so much time planning what to eat*,* how to purge*,* calculating calories… I had no energy left to study or do anything else. My grades just kept dropping”* (P8).

#### Factors contributing to deterioration

Several factors were found to exacerbate participants’ symptoms over time, including interpersonal conflict, external stressors, social misunderstanding, and limited awareness of EDs.

*Interpersonal conflicts* Interpersonal ruptures—such as family discord or romantic breakups—were frequently cited as triggers for worsening symptoms. These events intensified anxiety and hopelessness, leading individuals to rely more heavily on disordered eating for emotional regulation.

*“Especially when I was hit hard emotionally—by a breakup*,* or being misunderstood by my family—those were the worst times. I would get anxious*,* worried*,* and just… hopeless. Like I was falling into a deep abyss”* (P14).

*External stressful events* External stressors, such as academic pressure, work challenges, or financial difficulties, frequently contribute to symptom escalation. Participants described turning to food as a coping mechanism to manage or escape overwhelming stress.

*“When the pressure became overwhelming or things weren’t going well*,* I used food as a way to release that stress” (P7).*

*Social misunderstanding and denial* Participants consistently expressed that their symptoms were misunderstood or denied by people around them—including family, peers, and even healthcare providers. In the Chinese context, where mental health remains highly stigmatized, such invalidation deepened feelings of shame and social withdrawal.

*“No one around me took it seriously. They just thought I was being dramatic. So I stopped talking about it altogether”* (P5).

*Limited mental health literacy* Most participants had little awareness of EDs during the early stages of illness. Many misattributed their symptoms to physical or situational causes, leading to significant delays in recognition and care.

*“At the time*,* I thought I just had a cold or was a bit run-down. I was under so much pressure*,* but I didn’t think anything was really wrong. I didn’t know what EDs even were”* (P12).

This knowledge gap—reflective of broader deficiencies in mental health education—prevented early intervention and allowed symptoms to escalate.

#### Turning point: emergence of motivation for change

Despite the chronic and self-reinforcing nature of disordered eating behaviors, many participants described pivotal moments when the desire for change began to emerge. These shifts were often triggered by three main factors: the unbearable burden of the disorder, longing for a better future, and receiving acceptance and support from others.

*Recognition of harm and loss* For many, the initial spark of change arose from an increasing awareness of how severely EDs were impacting their body, mind, and life. Physical symptoms such as fatigue, menstrual irregularities, and pain served as visceral reminders that the disorder was taking a significant toll. In parallel, some participants described the emotional cost—estrangement from peers, academic setbacks, or the hollowing of daily life—as unacceptable losses that prompted reevaluation.

*“I don’t want to live like this anymore”* Extreme dissatisfaction with current life circumstances and expectations for future life are often the main driving forces for respondents’ change in motivation. This future-oriented perspective often planted the seeds of change.

*“I really felt that if I couldn’t succeed this time*,* I might as well die. It was like burning my bridges—I had no other path left. I had to try*,* to see if I could make it out. I couldn’t live like this forever*,* waking up every day thinking only about food. That kind of life felt utterly pathetic”* (P13).

*Receiving acceptance and support from others* Moments of connection—especially when participants disclosed their struggles and were met with empathy rather than judgment—were described as powerful motivators. These experiences countered the shame and isolation, and helped participants feel seen, valued, and capable of change.

*“When I told my friends about everything*,* and they didn’t judge me—instead*,* they supported me—I felt like maybe I wasn’t so alone. That made me want to try to get better”* (P1).

These insights underscore that motivation for change often arises not from external pressure, but from deeply personal realizations and relational experiences that affirm one’s value and potential for recovery.

### Coping

This theme captures the varied and evolving strategies participants used to cope with their EDs. Coping efforts fell into three domains: seeking professional help, receiving social support, and engaging in self-directed regulation (see Table 4).

**Table 4:** Coping

Subcategory	Focused Code	Number of participants	Frequency
Professional Help	Relief After Diagnosis.	5	8
	Medical and Hospitalization Experiences as “Relatively Failed”	10	28
	Counseling as Insufficient	8	16
Social Support	Acceptance and Proactive Understanding of EDs	6	16
	Companionship and Encouragement.	12	42
Self-Adjustment	Behavioral Strategies: Moving Away from Control	10	28
	Cognitive Shifts: Reframing Values and Self-Worth	6	13
	Emotional Transformation: Cultivating Self-Compassion	6	10

This table outlines the coping strategies participants used in managing their eating disorders, categorized into professional help, social support, and self-adjustment. The number of participants referencing each focused code and the frequency of its occurrence in interviews are listed

#### Seeking professional help

*Relief after diagnosis* Receiving a diagnosis helped alleviate participants’ guilt and self-blame by offering an explanation for their struggles and validating their experiences. For many, it fulfilled a deep desire to be seen and understood, fostering hope for recovery.

*“It’s a bit of a relief*,* like feeling seen. I really wanted someone to help me*,* but I couldn’t say it out loud. And at that moment*,* it felt like all my secretive behaviors could be understood” (P12).*

*Medical and hospitalization experiences as “relatively failed”* Several participants described inpatient or outpatient care as disappointing or even discouraging. These challenges were often attributed to clinicians’ dismissive attitudes, lack of tailored support, or treatment protocols that felt overly generalized and disconnected from their lived realities, leading to relapse after discharge.

*“I went to the hospital*,* but it felt like no one really understood. I didn’t feel seen or supported. It made me doubt whether recovery was possible”* (P6).

*Counseling as insufficient* While some participants sought psychotherapy, many found it insufficient to address the complexity of their condition. Short-term sessions were seen as superficial, unable to challenge entrenched beliefs about thinness, control, and self-worth.

*“I tried counseling*,* but I didn’t feel like it helped. I couldn’t talk myself out of wanting to be thin. It didn’t go deep enough”* (P11).

Participants’ accounts echo concerns raised in prior research, which argue that psychotherapy may be inefficient when it focuses primarily on individual-level symptoms without attending to the broader sociocultural and systemic forces shaping EDs [20, 36]. Pervasive weight stigma, the moralization of food and the body, and social scrutiny of women’s appearance can render recovery counter-cultural and reinforce the very beliefs that therapy seeks to challenge. These contextual pressures influenced participants’ sense of self-worth and their treatment by others, suggesting that effective intervention may require attention to both intrapsychic distress and the wider social dynamics in which EDs develop.

Additionally, others found it hard to build trust with therapists or felt misunderstood.

#### Social support

Participants frequently highlighted the importance of understanding and support from family and friends. Such support not only facilitated their coping with the illness but also instilled confidence and courage to face challenges.

*Acceptance and proactive understanding of EDs* For interviewees, the acceptance from those around them toward both their eating difficulties and themselves was vital.

*“It’s the greatest help and the best feeling when family and friends can accept me. They don’t see it as a terrible or shameful behavior; they want to accompany me to get better”* (P1).

Some participants shared that their family members actively sought out knowledge about EDs to better understand and support them, which was deeply moving and empowering.

*“I’m really grateful to my parents because they researched a lot of information for me”* (P5).

*Companionship and encouragement* Ongoing support through daily interactions—especially nonjudgmental presence during meals or emotional reassurance—helped ease anxiety and foster emotional resilience.

*“My mom told me not to worry and to eat as much or as little as I wanted. When she said this*,* I suddenly felt a lot more relaxed” (*P11).

Many participants also described the positive influence of their romantic partners, whose affirmation and emotional validation helped counteract body image anxieties and self-doubt.

*“My boyfriend told me that I’m beautiful*,* whether I weigh 130 or 150 pounds… and that no matter what*,* he would still love me”* (P13).

These forms of emotional support provided crucial reassurance and helped participants develop a more compassionate view of themselves.

#### Self-adjustment

Participants described a range of behavioral, cognitive, and emotional strategies they had developed or discovered independently. These self-initiated changes often emerged through trial and error, personal reflection, or in response to unmet needs within professional support systems.

*Behavioral strategies: moving away from control* Participants frequently reported modifying their daily habits to reduce the intensity of disordered eating patterns. Common strategies included introducing moderate exercise, abandoning strict food rules, and shifting focus to non-food-related activities.

*“I slowly let go of the black-and-white thinking about food—like good vs. bad food. Once I stopped labeling things*,* I could eat more freely and feel less afraid”* (P8).

*Cognitive shifts: reframing values and self-worth* Many reported reevaluating their values and redefining self-worth beyond appearance. This shift was linked to maturation and exposure to broader life experiences.

*“In the past*,* I equated being thin with being worthy. But now*,* I realize that I have other values—my personality*,* my work*,* my relationships. Those things matter more than numbers on a scale”* (P10).

This pattern aligns with broader research indicating that reframing personal values is a core component of ED recovery processes [20], [35]. Recent work also highlights how such shifts may involve reinterpreting cultural expectations shaped by intergenerational pressures or trauma [44].

*Emotional transformation: cultivating self-compassion* Several participants described learning to accept relapses and setbacks with greater patience, which helped sustain long-term recovery.

*“Now I take things more lightly. I’ve learned to go with the flow. If I relapse*,* it’s okay—I’ll get better slowly. I don’t pressure myself like before”* (P7).

Together, these coping strategies reflect a complex, iterative process involving both external support and internal growth.

### Theoretical model of the genesis, development, and coping of EDs

**Fig. 1:**
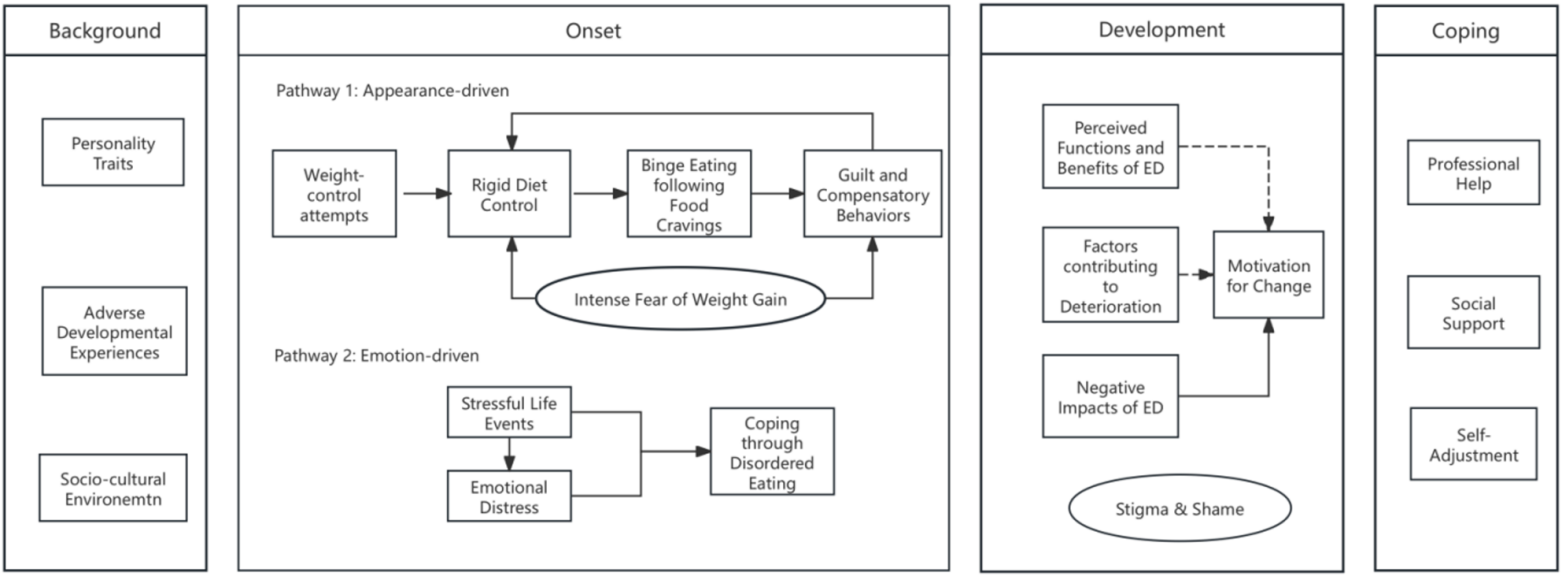
Theoretical model of eating disorders. A visual representation of the grounded theory model developed in this study, illustrating the relationships among risk factors, onset pathways, maintenance mechanisms, and change processes

Drawing on constant comparative analysis, a theoretical model was constructed to explain how EDs emerge, evolve, and are managed among young Chinese women. This model comprises four interrelated domains: risk background, onset pathways, developmental processes, and coping responses (see Fig. 1).

#### Risk background: vulnerability factors

Participants shared common vulnerability factors that heightened their susceptibility to EDs, including specific personality traits (e.g., perfectionism, low self-esteem), adverse developmental experiences (e.g., bullying, trauma), and sociocultural pressures (e.g., thin-ideal internalization, gendered appearance norms). These risk factors interacted to shape dysfunctional attitudes toward the body, food, and emotions, creating fertile ground for disordered eating.

#### Onset pathways: two distinct routes

Two distinct yet sometimes overlapping pathways to the onset of EDs were identified.

*Pathway 1: appearance-driven ED* Under societal and interpersonal pressure to achieve thinness, participants adopted restrictive eating habits. This often led to extreme hunger and uncontrollable binge eating, followed by feelings of shame and compensatory behaviors, forming a cyclical pattern maintained by fear of weight gain and temporary relief.

*Pathway 2: emotion-driven ED* Other participants developed EDs as coping mechanisms for stress, loneliness, or emotional dysregulation, independent of body image concerns. In the absence of healthy emotion regulation strategies, food became a means of escape or comfort, leading to habitual binge eating and emotional reinforcement.

#### Development: maintenance, deterioration, impact, and turning points

EDs were sustained by their perceived functions. First, individuals often receive positive feedback following weight loss, which strengthens their motivation to maintain a slender physique. Additionally, binge eating and purging may become maladaptive but significant means for emotional regulation and stress management, providing a sense of control, achievement, or self-worth. Finally, binge eating temporarily relieves individuals of hunger, satisfies their cravings for food, and subsequent purging behaviors alleviate their fear of weight gain, thus maintaining the disorder.

Several factors exacerbated symptoms over time, including interpersonal conflicts, external stressors, misunderstandings or denial from others, and the individuals’ own lack of ED knowledge. These factors, whether operating independently or in combination, exacerbated emotional distress and psychological pressure.

EDs severely impaired participants’ physical health (e.g., fatigue, organ dysfunction, menstrual irregularities), mental well-being (e.g., anxiety, depression, self-hatred), social functioning (e.g., isolation, avoidance of shared meals), and academic/work performance (e.g., loss of focus, reduced productivity).

Awareness of harm, combined with external support and emotional validation, catalyzed shifts in motivation for change, leading participants to seek help and consider recovery.

#### Coping: professional, others, and self

Coping strategies for eating disorders typically involve a combination of professional support, social support, and self-directed efforts.

Regarding professional support, individuals often experience some relief after receiving a diagnosis; however, many experienced treatment as insufficient. They felt that healthcare providers sometimes lacked adequate understanding or individualized care, and inpatient programs were viewed as generic, leading to relapse after discharge. Participants’ experiences also suggested that treatment often failed to address broader systemic and contextual factors underlying ED development and maintenance—such as sociocultural ideals, relational dynamics, and the social currency attached to thinness—which limited its perceived effectiveness.

Social support from significant others plays a crucial role in improving the situation. When family members or friends actively seek to understand the nature of EDs, acknowledge the existence of the problem, and provide support, companionship, and care, individuals with EDs are more motivated to face challenges and engage in the recovery process.

Self-adjustment is another key component of coping. Behaviorally, individuals adopted moderate exercise, loosened rigid food rules, and used distraction to reduce food preoccupation. Cognitively, they reframed perceptions of their problems, themselves, and the world in more adaptive ways. Emotionally, individuals practiced self-acceptance and compassion to ease distress and foster resilience.

## Discussion

This study developed a culturally grounded model of eating disorders (EDs) among young Chinese women, positioning individual experiences within broader sociocultural and psychological mechanisms. Through this theoretical model, the study captures the dynamic and multifaceted trajectories of ED experiences in this population. The model elucidates how culturally specific risk factors generate vulnerability, how the disorder emerges through distinct yet convergent pathways, how it is maintained or intensified over time, and how various coping resources contribute to change.

Previous research has emphasized sociocultural pressures [18], difficulties in emotion regulation [38, 50], reinforcement processes [12], body-related shame [46], and motivational dynamics [13]. Building upon this literature, the present study offers new insights into the lived experiences of Chinese women with EDs, illustrating how sociocultural and emotional processes intertwine in shaping illness trajectories. Specifically, our analysis identifies six interrelated themes: the moralization of aesthetic pressure, the functional role of ED behaviors in emotion regulation, reinforcement through social validation, vulnerabilities underlying weight fear, shame as a pervasive obstacle, and fragile yet transformative motivational shifts.

Taken together, these findings conceptualize EDs not merely as psychiatric syndromes but as culturally embedded conditions that evolve within specific moral, relational, and emotional contexts. This culturally informed framework provides a nuanced understanding of how EDs are constructed and maintained in non-Western settings and highlights the need for prevention and intervention strategies that are contextually sensitive and culturally responsive.

### Sociocultural context: aesthetic pressure as a structural force

Existing research has shown that sociocultural pressures such as social media exposure, peer comparison, and familial expectations are major predictors of body dissatisfaction and disordered eating across cultures [9, 18]. These influences extend beyond appearance ideals, embedding thinness and weight control within moral and social norms that define personal worth. Family evaluations of body weight often serve as one of the earliest channels through which sociocultural beauty standards are transmitted. Longitudinal evidence indicates that parent-perceived childhood overweight predicts later binge eating and purging behaviors through early adolescent concerns about eating, weight, and shape [4], suggesting that parental perceptions may initiate internalized monitoring long before disordered eating develops.

Previous studies on Chinese women with EDs have also revealed that young women face gendered scrutiny of their bodies and are compelled to engage in self-surveillance to gain familial and social approval (Holmes & Ma, 2024). Within this cultural climate, body monitoring and weight evaluation are further reinforced, as losing control over one’s body is perceived not only as an aesthetic failure but also as a moral lapse—a failure to meet family or societal expectations.

Building on this literature, the present study deepens understanding of the moralized and relational nature of body surveillance in China, emphasizing aesthetic pressure as a structural force shaping vulnerability to EDs. Participants described how appearance-related commentary had become normalized: family remarks were often framed as “caring concern,” while peer comments were treated as casual jokes, making body monitoring feel like an obligation. Being told to lose weight or praised for being slim was perceived not merely as personal feedback but as an expectation from loved ones. This discourse situates body management within moral expectations of self-discipline and social value, while weight gain was often interpreted as a lack of control or a source of disappointment.

These findings suggest that effective prevention requires culturally grounded strategies that move beyond media literacy to address family and peer discourse. In collectivist settings like China, involving parents and educators in redefining beauty standards and promoting body diversity is essential. Clinicians and schools should work collaboratively to decouple weight from moral value and foster supportive, nonjudgmental communication. Ultimately, addressing eating disorders demands not only individual-level education but also community-level transformation in attitudes toward body image.

### Two distinct routes: sociocultural vs. emotional pathways

Within this sociocultural context, two distinct yet overlapping pathways emerged in the development of eating disorders among young Chinese women.

*Appearance-driven route*

Aligned with the sociocultural model of eating disorders [62] and objectification theory [26, 56], this pathway shows how thin-ideal internalization and body dissatisfaction lead to restrictive dieting, which often escalates into binge–purge cycles sustained by shame and fear of weight gain. Meta-analytic evidence confirms that thin-ideal internalization and self-objectification predict disordered eating via body surveillance and shame [45, 55, 63].

Our study extends these models by revealing how the moralization of thinness amplifies such processes in the Chinese context. Participants described thinness as a marker of virtue and discipline, while weight gain signified moral weakness and social failure. Within collectivist norms, body size thus carries ethical meaning, intertwining appearance control with relational expectations. Situating the onset of EDs in this moral discourse refines existing Western frameworks through a culturally grounded lens.

*Emotion-driven route*

Emotion regulation difficulties also play a central role in EDs [12, 38, 50, 52]. Participants framed disordered eating as a maladaptive coping strategy providing brief relief: binge eating reduced distress, whereas restriction or purging restored control. These findings align with emotion regulation models (Aldao et al., 2010), which view ED behaviors as functional yet harmful responses to overwhelming affect.

In Chinese cultural contexts, where emotional expression is discouraged, food often becomes a primary means of affect regulation, reinforcing reliance on eating behaviors. Integrating emotion regulation with sociocultural constraints, this study highlights the need for culturally sensitive interventions that strengthen adaptive coping and address barriers to emotional disclosure.

*Intersection and clinical implications*

These pathways were not mutually exclusive. For some participants, appearance-based pressures and emotional distress were intertwined—body dissatisfaction intensified negative affect, and emotional upheaval, in turn, reinforced body concerns. Recognizing these dual routes underscores the heterogeneity of ED onset and cautions against uniform conceptualizations.

Clinically, appearance-driven cases may benefit from interventions targeting thin-ideal internalization, media literacy, and familial discourse, whereas emotion-driven cases may require culturally sensitive approaches that enhance emotional awareness, adaptive coping, and interpersonal support.

Acknowledging both sociocultural and emotional pathways enables a more nuanced understanding of ED development and informs flexible, individualized strategies for prevention and treatment.

### Functional reinforcements and social validation

Participants reported that their eating disorder behaviors were sustained by perceived functions and reinforcements. Early weight loss often drew praise from family, peers, or social networks, reinforcing motivation to stay thin. Over time, binge eating and purging became maladaptive yet rewarding strategies to regain control, reduce distress, or affirm self-worth.

These findings align with evidence that positive reinforcement for weight loss can trigger and maintain restrictive eating [33] and that parental comments about weight are strongly associated with body dissatisfaction and disordered cognitions [17]. In digital environments, thinspiration content promotes thinness and restriction, while social media interactions amplify disordered eating through social comparison and ideal internalization [3, 18]. Ecological studies further show that binge eating temporarily alleviates negative affect and restriction enhances perceived control [12, 57]. In China, where thinness is moralized as a symbol of self-discipline and social worth, such reinforcements may be especially powerful [31].

Recognizing these reinforcement processes has important clinical implications. Treatment should help patients replace disorder-based validation with healthier sources of self-worth. Therapists can encourage engagement in meaningful activities and relationships that foster competence and belonging independent of weight, while families can shift praise away from appearance to reduce inadvertent reinforcement. Strengthening intrinsic self-esteem over time may lessen reliance on disordered behaviors for reward and control.

### Beneath weight fear: psychological and relational vulnerabilities

This study found that fear of weight gain was closely tied to deeper anxieties about failure, rejection, and loss of control—reflecting unmet psychological needs and fragile self-esteem. This aligns with recent evidence that fear of fatness and body-related anxiety are among the strongest predictors of restrictive eating in young women [9]. Such fears often signify underlying vulnerability rather than mere preoccupation with appearance.

From an objectification perspective, women in appearance-focused cultures may internalize external gaze and evaluate their bodies as objects, amplifying shame and weight-related anxiety [54]. This mechanism appears especially salient in China, where body control is moralized as a sign of discipline and social worth. Network analyses of Chinese samples similarly indicate that fear of weight gain, emotional dysregulation, and perfectionism are central features maintaining disordered eating [71].

Together, these findings underscore that interventions must go beyond body image to address the symbolic meanings attached to weight and appearance. Therapeutic work should explore how fears of inadequacy, rejection, and loss of control are displaced onto the body, and help patients cultivate alternative sources of self-worth and belonging. Enhancing self-compassion and challenging self-objectification may be particularly effective in reducing the psychological hold of weight fear.

### Shame as a pervasive and relational obstacle

Recent evidence shows that shame is closely tied to the onset and maintenance of EDs, with body- and eating-related shame emerging as strong predictors [11, 46]. Momentary assessments further indicate that increases in shame can immediately precede disordered behaviors such as binge eating and body checking [47]. Shame and stigma also remain major barriers to help-seeking, restricting disclosure and delaying treatment [2].

In the Chinese context, shame functioned not only as internalized self-disgust but also as a relational and moral experience shaped by family judgments and collective expectations of bodily control. This perspective extends prior research by framing shame as both intrapsychic and structural, deepening its role in concealment and chronicity. Unlike guilt, which is behavior-specific and reparative, shame eroded participants’ sense of self, reinforced isolation, and discouraged help-seeking. These findings highlight the need for interventions that explicitly address shame—such as compassion-focused therapy—while challenging the cultural moralization of body restraint and emotional suppression.

### A fragile turning point: motivation for change

Participants’ recovery trajectories hinged on the emergence of autonomous motivation, often catalyzed by cumulative distress or moments of genuine understanding from others. For individuals with chronic or severe eating disorders, regaining autonomy and agency is critical for initiating change. When treatment feels overly controlling, motivation weakens; autonomy-supportive contexts, by contrast, foster engagement and persistence [13].

Consistent with self-determination theory, sustained recovery depends on fulfilling basic psychological needs for autonomy, competence, and relatedness [25, 53]. In this study, relational acceptance—being “seen” and supported without judgment—proved transformative, showing how authentic connection can reignite intrinsic motivation. When external expectations are internalized and aligned with personal values, motivational shifts become more enduring.

These findings highlight the importance of interventions that strengthen patients’ autonomy and self-compassion while engaging supportive others as recovery allies. Approaches such as motivational interviewing and SDT-informed therapy can help patients clarify personally meaningful goals, transforming fragile motivation into a stable foundation for long-term change.

### Strengths and limitations

This study has several methodological strengths. It is among the few qualitative inquiries addressing eating disorders among young Chinese women using a constructivist grounded theory framework. The study’s design allowed participants’ lived experiences to guide theory development, ensuring that the resulting model was grounded in their own meanings and sociocultural contexts. The use of multiple strategies—such as memo writing, peer debriefing, and reflexive analysis—enhanced the credibility and transparency of the findings.

Despite these contributions, several limitations should be noted. As a small-scale qualitative study, the sample was relatively homogeneous: most participants were highly educated, urban, and recruited online. This limits the transferability of the findings to women from different educational, regional, or socioeconomic backgrounds, as well as to those who have not accessed diagnosis or treatment. In addition, all participants were self-reporting current or past ED diagnoses, and their accounts were retrospective; recall bias and self-presentation concerns may have shaped how experiences were narrated. Finally, although reflexive strategies such as memo writing, peer debriefing, and supervision were applied, researcher subjectivity may still have influenced data interpretation—an inherent feature of constructivist grounded theory.

### Future directions

Future research could build on these findings in several ways. Expanding recruitment to include participants from more diverse demographic and socioeconomic backgrounds would enhance the transferability and inclusiveness of results. Quantitative or mixed-methods studies could test and refine the proposed theoretical model, improving its empirical robustness. Longitudinal designs tracking illness trajectories over time would provide insight into chronicity, relapse, and recovery processes. Finally, applied research should examine how culturally grounded models can inform targeted interventions in clinical practice, helping to develop more effective, shame-sensitive approaches for individuals with eating disorders in China and other collectivist contexts.

## Conclusion

This study employed a constructivist grounded theory approach to examine the lived experiences of young Chinese women with eating disorders, yielding a culturally embedded model of how these disorders emerge, develop, and are managed. Drawing on in-depth interviews with 19 participants, the analysis identified two primary entry pathways—concerns about body weight and struggles with emotional distress—and revealed how disordered eating behaviors serve functional roles in regulating emotions, affirming self-worth, and negotiating relational dynamics.

Importantly, this study sheds light on how deeply relational emotions such as shame, combined with rigid cultural expectations and aesthetic norms, contribute to both the emergence and persistence of eating disorders in this population. It also illustrates the critical role of change motivation and social support—particularly the healing potential of being seen, understood, and accepted by others—in sustaining recovery efforts.

By foregrounding the perspectives of Chinese women, this study offers a culturally situated account that may complement existing ED frameworks and broaden understandings of how these disorders are experienced outside Western contexts. While the model developed here is not intended to be universal, it underscores the value of attending to sociocultural influences when interpreting ED experiences and may help inform more culturally responsive clinical practices. Future research with larger and more diverse samples is needed to further examine how cultural contexts shape ED trajectories and to refine theoretical models accordingly.

## Supplementary Information

Below is the link to the electronic supplementary material.

Supplementary Material 1

Supplementary Material 2

## Data Availability

Due to the sensitive nature of qualitative data and to protect participants’ privacy, interview transcripts are not publicly available. De-identified excerpts may be available from the corresponding author upon reasonable request.
