# Supplementary material for: Between shame, control, and survival: a grounded theory study of eating disorders among young Chinese women
Source: J Eat Disord. 2026 Mar 31;14:109. doi: 10.1186/s40337-025-01510-9 (PMC13162519; doi:10.1186/s40337-025-01510-9)
Supplement: Supplementary file 1 — Supplementary Material 1 [file 40337_2025_1510_MOESM1_ESM.pdf]

**Additional file 1 – COREQ (Consolidated Criteria for Reporting Qualitative Research) Checklist**

Title of study: *Between Shame, Control, and Survival: A Grounded Theory Study of Eating Disorders Among Young Chinese Women*

Reference: Tong, A., Sainsbury, P., & Craig, J. (2007). Consolidated criteria for reporting qualitative research (COREQ): a 32-item checklist for interviews and focus groups. *International Journal for Quality in Health Care, 19*(6), 349–357.

| Domain / Item | Reported in manuscript (page/section) | Details for this study |
|---|---|---|
| **Domain 1: Research team and reflexivity** | | |
| 1. Interviewer | Methods 2.3 | QT conducted all interviews. |
| 2. Credentials | Tile page | QT, JJ – M.A.; SF – Ph.D. |
| 3. Occupation | Tile page | During data collection, QT and JJ were graduate students in psychology; SF was a lecturer in the Faculty of Psychology at Beijing Normal University. |
| 4. Gender | Method 2.6 | All researchers were female. |
| 5. Experience and training | Methods 2.1; Methods 2.6 | QT and JJ completed formal qualitative research training through the MAP (Master of Applied Psychology) program at Beijing Normal University and received supervision during data collection and analysis.<br>SF holds a Ph.D. in Counseling Education and Supervision, has extensive experience in qualitative and mixed-methods research, and provided methodological |

| | | |
|---|---|---|
| | | supervision and oversight throughout the project. |
| 6. Relationship established | Method 2.2 | No prior relationship with participants. |
| 7. Participant knowledge of the interviewer | / | Participants were informed that the interviewer was a psychology researcher studying women's experiences of eating disorders. |
| 8. Interviewer characteristics | Methods 2.6 | The interviewer (QT) shared gender and cultural background with participants and maintained reflexive notes on how her perspectives might shape data collection and interpretation. Reflexivity was discussed regularly in supervision meetings. |
| **Domain 2: Study design** | | |
| 9. Methodological orientation and theory | Methods 2.1 | Constructivist grounded theory (Charmaz, 2008). |
| 10. Sampling | Methods 2.2 | Purposive and theoretical sampling were used to recruit young Chinese women with current or past eating disorder diagnoses, ensuring diversity in illness type, duration, and recovery stage. |
| 11. Method of approach | Methods 2.2 | Participants were approached through online recruitment posts on university mental health platforms, social media, and peer referrals. Contact occurred via texts and phone-calls. |
| 12. Sample size | Methods 2.2 | N = 19 (five pilot interviews, fourteen formal interviews). |
| 13. Non-participation | Method 2.3 | None refused or withdrew after giving consent. |
| 14. Setting of data collection | Methods 2.3 | Interviews conducted online. |

| | | |
|---|---|---|
| 15. Presence of non-participants | Method 2.3 | No. |
| 16. Description of sample | Methods 2.2;Table 1. Pilot Interview Participant Demographics; Table 2. Formal Interview Participant Demographics | Participants were young adult Chinese women aged 18–30 with either a current or past diagnosis of anorexia nervosa, bulimia nervosa, and/or binge-eating disorder (DSM-5 criteria). They represented varied illness severities, durations, and recovery stages. |
| 17. Interview guide | Methods 2.3 | Yes. A semi-structured interview guide was developed based on the study's aims and relevant literature. It included four main domains: illness onset, maintenance and emotional regulation, sociocultural and relational influences, and coping or recovery. Open-ended questions encouraged participants to share freely. The guide was reviewed by experts in clinical psychology and qualitative research and refined through five pilot interviews, after which minor revisions were made for clarity, sensitivity, and flow. |
| 18. Repeat interviews | Methods 2.3 | No; each participant interviewed once. |
| 19. Audio/visual recording | Methods 2.3 | Audio recorded with participants' consent. |
| 20. Field notes | Methods 2.3 | Yes. The primary interviewer (QT) wrote reflective field notes and analytic memos after each interview to capture contextual observations, emotional tone, and emergent ideas. |
| 21. Duration | Methods 2.3 | 60–90 minutes. |

| | | |
|---|---|---|
| 22. Data saturation | Methods 2.3 | Yes. Data collection continued until theoretical saturation was reached. After twelve formal interviews, no new conceptual categories emerged; two additional interviews were conducted to confirm saturation. |
| 23. Transcripts returned | Methods 2.5 | No; participants did not review transcripts to preserve anonymity. |
| **Domain 3: Analysis and findings** | | |
| 24. Number of data coders | Methods 2.4 | Two researchers (QT and JJ) were involved in the coding process. The first author (QT) conducted the primary coding of all interview transcripts, while the second author (JJ) independently coded the majority of transcripts. Coding discrepancies were discussed and resolved through consensus under the supervision of the senior author (SF). |
| 25. Description of the coding tree | Methods 2.4 | Yes. Data were analyzed through four iterative stages following Charmaz's constructivist grounded theory framework: (1) initial coding, (2) focused coding, (3) axial coding, and (4) theoretical coding. |
| 26. Derivation of themes | Methods 2.4 | Themes were derived inductively from the data. Coding and theme development followed constant comparison and iterative refinement consistent with grounded theory methodology, allowing categories to emerge directly from participants' narratives. |
| 27. Software | Methods 2.4 | NVivo 12. |
| 28. Participant checking | Methods 2.5 | Partial member checking was conducted. Three participants representing different diagnostic and recovery profiles reviewed thematic summaries and confirmed that they accurately reflected their lived experiences. |

| | | |
|---|---|---|
| 29. Quotations presented | Results | Yes. Direct quotations were used throughout the Results section to illustrate key categories and subthemes. Each quotation was anonymized and labeled to preserve confidentiality while ensuring transparency. |
| 30. Data and findings consistent | Results | Yes. The reported findings were firmly grounded in participants' words and illustrated with representative quotations. |
| 31. Clarity of major themes | Results | Yes. Four major themes were clearly presented in the Results: (1) Risk factors (personality traits, adverse experiences, sociocultural environment); (2) Onset pathways (weight-concern vs. emotion-driven routes); (3) Development and change (functions of EDs, shame, deterioration, and motivation for change); and (4) Coping strategies (professional help, social support, self-adjustment). Each theme was support by participant quotations, summarized in Tables 3–6, and integrated in a final theoretical model (Figure 1). |
| 32. Clarity of minor themes | Results | Yes. Variations and overlaps between the two onset pathways and among coping experiences were described, showing mixed trajectories and diverse impacts. These differences were used to refine each theme and to ground the integrative model. |

Summary: This study met COREQ reporting standards for qualitative interviews. It ensured transparency in researcher reflexivity, methodological rigor, and analytic process within a constructivist grounded theory framework.
