# Supplementary material for: Between shame, control, and survival: a grounded theory study of eating disorders among young Chinese women
Source: J Eat Disord. 2026 Mar 31;14:109. doi: 10.1186/s40337-025-01510-9 (PMC13162519; doi:10.1186/s40337-025-01510-9)
Supplement: Supplementary file 2 — Supplementary Material 2 [file 40337_2025_1510_MOESM2_ESM.pdf]

Additional file 2 – Semi-Structured Interview Guide

Study title: *Between Shame, Control, and Survival: A Grounded Theory Study of Eating Disorders Among Young Chinese Women*

Purpose: To explore how young Chinese women experience, interpret, and cope with eating disorders, and how sociocultural and emotional factors shape their illness and recovery processes.

Development and validation of the guide

The semi-structured interview guide was developed by the research team based on prior literature, research aims, and clinical insights into eating disorders. It covered four broad domains: illness onset, maintenance mechanisms, psychosocial and cultural influences, and coping or recovery strategies. The draft guide was reviewed by experts in clinical psychology and qualitative research for clarity and sensitivity, then pilot-tested with five participants who met the study criteria. Minor revisions were made to improve flow, emotional safety, and thematic coverage. The final guide was used for all 14 formal interviews.

Opening statement (used before each interview)

Hello, and thank you for taking part in this interview. I'm a graduate student in Applied Psychology at Beijing Normal University. This conversation focuses on your personal experiences related to eating, emotions, and body image. The interview will take about 60–90 minutes.

Please feel free to share whatever feels meaningful to you—there are no right or wrong answers. Everything you share will remain confidential and used only for research purposes. You can pause or skip any question at any time. With your permission, the interview will be audio-recorded to help us capture your words accurately.

If you're ready, we may begin.

Section 1: Background Information

1. Could you start by telling me a little bit about yourself?

2. What made you interested in participating in this study?

Section 2: Illness Onset and Development

3. Could you describe how your eating-related experiences began and how they have changed over time?

    (1) When did you first notice that something might be a problem?
    (2) Looking back, when do you think these concerns first appeared?
    (3) How did your eating behaviors develop, and what might have contributed to them?
    (4) Are there aspects of your upbringing or life experiences that you feel are connected to your eating or body image?
    (5) Could you tell me about the process of receiving a diagnosis (if applicable)? How did you feel about it?
    (6) How would you describe your current situation related to eating or body image?
    (7) What are your hopes or expectations for the future?

Section 3: Impacts and Influencing Factors

4. How has the eating disorder affected your life—such as your relationships, studies or work, and emotional well-being?

    (1) Were there any aspects that, in some ways, helped you, or that made things more difficult?

5. What factors do you think have made your situation worse, and what factors have helped?

    (1) What has been most helpful for you personally?
    (2) Looking back, what kind of support would you have liked to receive?

    (This section covers individual, interpersonal, and sociocultural influences.)

Section 4: Coping and Recovery

6. Could you talk about the ways you've tried to cope with or recover from your eating difficulties?

(1) What kinds of attempts have you made to manage or change your eating behaviors?

(2) Which strategies worked, and which did not?

Section 5: Meaning-Making and Reflection

7. How do you understand or make sense of your eating disorder now?

8. If you could say something to other young women who may be struggling—or to those who haven't experienced these issues—what would you want them to know?

Closing statement

Thank you for sharing your experiences today. Some of these topics can be emotional, so please take some time to rest or reach out to your supports afterward. If you would like, I can share a list of local mental health services and online resources.

Implementation details

• Interview length: 60–90 minutes per session

• Setting: Online via secure video platform or private conference room

• Language: Mandarin Chinese

• Recording: Audio-recorded with consent

• Pilot testing: Conducted with 5 participants before formal data collection

• Final version used for: 14 formal interviews (N = 19 total participants including pilot)

• Interviewer: Graduate student in clinical and counseling psychology with prior experience in counseling and crisis intervention

• Crisis protocol: Participants could pause or withdraw at any time. Supportive responses were provided when needed, and each participant received a list of mental health resources at the end of the interview.
